# An exploratory study on the metagenomic and proteomic characterization of hypothyroidism in the first half of pregnancy and correlation with Th1/Th2 balance

**DOI:** 10.3389/fimmu.2025.1500866

**Published:** 2025-05-15

**Authors:** Chenchen Zhang, Yajuan Xu, Miao Zhang, Jingjing Li, Zongzong Sun, Yixin Wang, Pengkun Lin

**Affiliations:** Department of Obstetrics and Gynecology, Third Affiliated Hospital of Zhengzhou University, Zhengzhou, China

**Keywords:** first half of pregnancy, hypothyroidism, metagenomics sequencing, proteomics, gut microbiota

## Abstract

**Objective:**

To explore the gut microbiota and proteomic characteristics of hypothyroidism in the first half of pregnancy (referred to as hypothyroidism in the first half of pregnancy) and its association with Th (T helper cells, Th)1/Th2 balance using metagenomics combined with proteomics.

**Methods:**

Stool and blood samples were collected from 20 hypothyroid (hypothyroidism group) and normal pregnant women (normal group) in the first half of pregnancy. Flora and proteomic characteristics were analyzed using metagenomics sequencing and 4D-DIA proteomics. Th1 and Th2 cells were quantified, and cytokine levels were measured using cellular micro-bead arra. The enzyme-linked immunosorbent test (ELISA) was utilized to assess differential proteins.

**Results:**

(1) Metagenomic sequencing revealed distinct microbial profiles: The β-diversity of gut microbiota was diminished in the hypothyroidism group (p < 0.05). LEfSe analysis identified *Phocaeicola vulgatus* and *Bacteroides fragilis* enriched in the hypothyroidism group (p<0.05), and Kyoto Encyclopedia of Genes and Genomes(KEGG) analysis showed significant enrichment in pathways related to peptidoglycan biosynthesis and glycerol ester metabolism.(2) Proteomic analysis demonstrated downregulation of Diacylglycerol Kinase Kappa (DGKK) and P05109|S10A8(S10A8) proteins in the hypothyroidism group, with marked enrichment in the KEGG pathways for vascular smooth muscle contraction and phosphatidylinositol signaling. (3) ELISA validation confirmed that the proteins DGKK and S10A8 were downregulated in pregnant women in the hypothyroidism group.

**Conclusion:**

Increased *P. vulgatus* and *B. fragilis*, decreased DGKK and S10A8 proteins, and a left shift in the Th1/Th2 balance in patients with hypothyroidism in the first half of pregnancy may be associated with the development of the disease.

## 1 Background

Hypothyroidism in pregnancy is a common endocrine disorder (1). Moreover, it increases the risk of adverse pregnancy outcomes such as miscarriage, gestational hypertension, and fetal malformations (2). We generally define the gestational age of less than 20 weeks as the first half of pregnancy (3). Notably, maternal and fetal demand for thyroid hormones increases during pregnancy, and the fetus is unable to synthesize thyroid hormones before 20 weeks of gestation (2). Therefore, hypothyroidism in the first half of pregnancy has a profound effect on fetal growth and development (2).The gut microbiota is recognized as the second-largest gene pool in humans. It provides essential metabolites and hormones, prevents invasion by pathogenic microorganisms, and is closely related to immune system disorders (4, 5). The gut microbiota affects thyroid homeostasis through multiple mechanisms and plays a role in the development of hypothyroidism during pregnancy (6, 7). Our group previously demonstrated that gut microbiota disorders exist in patients with hypothyroidism during the first half of pregnancy through 16SrRNA gene sequencing (8–10). Serum proteomics in women with hypothyroidism during the first half of pregnancy is underexplored, with few studies addressing this period. Metagenomics enables species-level annotation and analysis of gut microbial genes, functions, and metabolic pathways, revealing their biological significance. Proteomic methods screen for biomarkers and investigate disease pathogenesis.

In the first half of pregnancy, the Th1/Th2 balance shifts from Th2 to Th1 dominance by late pregnancy (11). Th1/Th2 imbalances, influenced by gut microbiota disorders, regulate inflammatory cytokine secretion and contribute to immune dysfunction, potentially altering inflammatory protein expression and impacting autoimmune disease development (12–14). Despite limited research, this study examines gut microbiota and proteomic characteristics in hypothyroid pregnant women, analyzing changes and associations with Th1/Th2 balance using metagenomic and proteomic approaches.

## 2 Materials and methods

### 2.1 Research objectives

From July to August 2024, pregnant women receiving regular perinatal care were selected at the Third Affiliated Hospital of Zhengzhou University’s outpatient obstetrics clinic. Twenty women in their first trimester with hypothyroidism who met the inclusion criteria comprised the hypothyroidism group, and 20 with normal pregnancies formed the normal group.

Inclusion Criteria: 1) thyroid function during pregnancy in accordance with the 2022 Guidelines for the Management of Thyroid Disease Prevention and Control in Pregnancy and Childbirth (1) and the reference range of hypothyroidism in pregnancy (TSH >4.0 mIU/L and FT4 <12 mIU/L) established by the Department of Laboratory of the Third Affiliated Hospital of Zhengzhou University. 2) The week of pregnancy was less than 20 weeks.

Exclusion criteria: 1) age below 18 or above 35 years; 2) pregnancy with complications; 3) use of artificial insemination or assisted reproductive technology; 4) multiple pregnancies (twins or more); 5) severe anxiety or depression; 6) history of circulatory, digestive, or other medical conditions, or past gastrointestinal surgery; 7) chronic use of antibiotics or medications regulating gut microbiota; 8) current use of anti-thyroid or thyroid replacement medications; 9) presence of liver disease, malignant tumors, or other severe systemic conditions; 10) ongoing infection or history of chronic inflammation and autoimmune diseases.

Ethics Statement: This study was approved by the Medical Ethics Committee of the Third Affiliated Hospital of Zhengzhou University, China. All enrolled members voluntarily participated and signed the informed consent forms.

### 2.2 Stool and blood sample collection and storage

All patients had their feces collected within 24 hours following the diagnosis of hypothyroidism in pregnancy. Prior to sample collection, they were instructed to ensure that the feces did not contact the bedpan, were free from urine contamination, and were collected from the mid-feces using a sterile spoon. The collected samples were then placed in sterile 2.0 mL tubes, transported to the laboratory within 2 hours, and stored at -80°C until further processing.

Blood samples were simultaneously collected, with all pregnant women fasting for 8–12 hours beforehand. Two 5 mL blood specimens were drawn from the elbow vein using a sterile syringe needle, deposited into Ethylenediaminetetraacetic acid (EDTA) and sodium heparin anticoagulation tubes, and immediately refrigerated at 4°C. The blood samples in EDTA tubes were centrifuged at 4°C, 2000 rpm for 10 minutes within 2 hours of collection. Subsequently, the upper serum layer was aspirated with a sterile pasteurized pipette and stored in a freezing tube at -80°C.

### 2.3 Data collection

Data on age, body mass index (BMI), gestational week, and serum-free T4 (FT4), thyroid-stimulating hormone (TSH), fasting glucose (GLU), hemoglobin (HGB), and hypersensitive C-reactive protein (hs-CRP) levels were collected from all pregnant women at enrollment.

### 2.4 Cytokine detection

Interleukin (IL)-2, IL-6, IL-10, and tumor necrosis factor-α(TNF-α) levels were measured using a Human Cytokine Kit (China Jiangxi Sage Biotechnology Co.) based on flow fluorescence technology. The kit contains microspheres encapsulating antibodies specific to IL-2, IL-6, IL-10, and TNF-α. The capture microsphere mixture is mixed with 25 μL of serum, and then the mixture is incubated with 25 μL of fluorescently labeled detection antibody for 2.5 h at 20-25°C under dark conditions. The beads were washed and resuspended in PBS, the samples were analyzed using flow cytometry, and the data were recorded. The samples were analyzed, and data were recorded using a flow cytometer (BD FACSC cantoII, USA) and corresponding software (BD FACSDiva Software, version 8.0.2) (15, 16).

### 2.5 Flow cytometry

Th1 and Th2 cells were quantified using intracellular cytokine staining after peripheral blood collection from pregnant women in sodium heparin anticoagulated tubes. 200ul of anticoagulated whole blood was added to each flow-through tube separately, and the dissolved stimulants (phorbol myristate acetate (50ng/mL), ionomycin (1mg/mL), and brefeldin A (10 mg/mL) were added to the flow-through tubes, and the tubes were incubated for 4 hours at 37°C in 5% CO2. followed by the addition of APC-CY7-CD3 (0.2mg/ml) and FITC-CD4 (0.2mg/ml) antibodies for cell surface labeling. Cells were incubated for 20 minutes in a light-proof environment, erythrocyte lysate was added, and the cells were incubated for 25 minutes. Cells were centrifuged at 500 g at 4°C, the supernatant was discarded, and a fixed membrane-breaking solution (Preparation of 1× Perm/Wash Buffer: The 10× Perm/Wash Buffer was diluted with deionized distilled water (ddH2O) at a volumetric ratio of 1:9 to achieve the desired 1× working concentration.) was added. Following a 30-minute incubation in darkness, cells were stained with PE-IFN-γ (0.2mg/ml) and PE-CY7-IL-4 (0.2mg/ml) antibodies for 30 minutes. After washing, cells were resuspended in phosphate buffer (PBS 2 ml) and analyzed via flow cytometry using FlowJo software (Tree Star, Ashland, OR, USA, version10.8.1). All antibodies were sourced from BD Biosciences (Franklin Lakes, NJ, USA).

**Figure 1:**
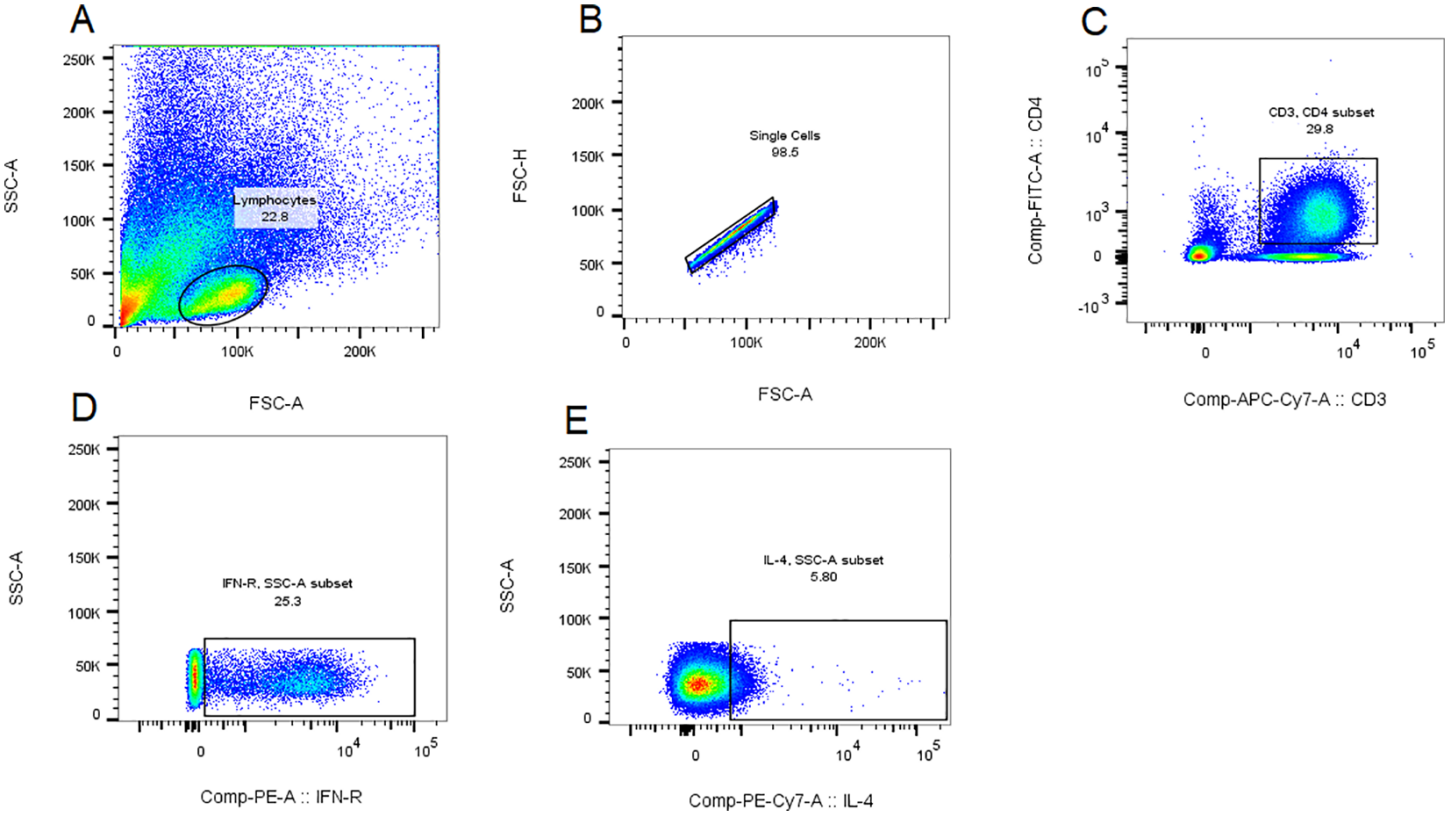
Th1 and Th2 cell gating strategy. The gating strategy was implemented following compensation controls. Initial lymphocyte population identification was performed based on cellular characteristics, utilizing forward scatter (FSC) and side scatter (SSC) parameters plotted on the x-axis and y-axis, respectively **(A)**. Subsequent to this primary gating, single-cell populations were isolated through the exclusion of doublets, achieved by plotting forward scatter height (FSC-H) against forward scatter area (FSC-A) on the y-axis and x-axis, respectively **(B)**. CD4+ T cell populations were then specifically gated using APC-CY7 and FITC fluorescence parameters on the x-axis and y-axis **(C)**. For quantitative analysis of T helper cell subsets, Th1 and Th2 cell populations were identified through the detection of intracellular cytokines, with IFN-γ and IL-4 expression plotted against SSC on the y-axis and x-axis, respectively, representing the proportions of CD3+CD4+IFN-γ+ Th1 cells and CD3+CD4+IL-4+ Th2 cells **(D, E)**. All cellular proportions and corresponding graphical representations were systematically recorded, with data analysis performed using FlowJo software (Tree Star, Ashland, OR, USA, version10.8.1).

Th1 and Th2 Cell Gating Strategy:The gating strategy was implemented following compensation controls. Initial lymphocyte population identification was performed based on cellular characteristics, utilizing forward scatter (FSC) and side scatter (SSC) parameters plotted on the x-axis and y-axis, respectively (**Figure 1A**). Subsequent to this primary gating, single-cell populations were isolated through the exclusion of doublets, achieved by plotting forward scatter height (FSC-H) against forward scatter area (FSC-A) on the y-axis and x-axis, respectively (**Figure 1B**). CD4+ T cell populations were then specifically gated using APC-CY7 and FITC fluorescence parameters on the x-axis and y-axis (**Figure 1C**). The percentage contents of Th1 and Th2 cells, represented by CD3+CD4+IFN-γ+ for Th1 cells and CD3+CD4+IL-4+ for Th2 cells, were displayed with IFN-γ and IL-4 as the abscissa and SSC as the ordinate, respectively (**Figures 1D, E**). All cellular proportions and corresponding graphical representations were systematically recorded, with data analysis performed using FlowJo software (Tree Star, Ashland, OR, USA, version10.8.1).

### 2.6 Metagenomics sequencing and data analysis

Total DNA was extracted from 0.20 g of fecal specimens using the MagPure Stool DNA KF Kit B (cat. no.MD511, Magen, China). To minimize the interference of host sequences in DNA extraction and subsequent analysis, the Bowtie2 software (version 2.4.4, https://github.com/BenLangmead/bowtie2) (17) was employed during the extraction process to remove sequences aligned to the host genome, thereby reducing the impact on microbial analysis. 1 μg of genomic DNA was then sheared ultrasonically using a Covaris instrument to produce 300 bp fragments for sequencing on the DNBSEQ platform (BGI, Shenzhen, China) using cPAS. The resultant fasta format sequences underwent quality control and assembly with Fastp and MEGAHIT software (version 1.1.2, https://github.com/voutcn/megahit) (18), respectively. Gene sets were annotated to the KEGG database (https://www.genome.jp/kegg/) via Diamond software (version 2.0.13, https://github.com/bbuchfink/diamond) (19). Non-redundant gene sets were clustered using CD-HIT (version 4.8.1, https://github.com/weizhongli/cdhit) (20) and compared to the NR database (https://ftp.ncbi.nlm.nih.gov/blast/db/FASTA/) using BLAST (version 2.2.28, http://blast.ncbi.nlm.nih.gov/Blast.cgi) (21) Statistical analysis was performed using the Wilcoxon rank-sum test and LDA.

### 2.7 Proteomics analysis and data processing

Mass spectrometry data from 40 samples were collected using a TimsTOF Pro instrument in the data-independent acquisition (DIA) mode. The experimental procedure was as follows: protein extraction and enrichment were performed using a C18 column. After enrichment and quality control, 20 μg of peptides from each sample were combined and eluted using a Shimadzu LC-20AB liquid phase system. Liquid phase separation was performed with a 5 μm 20 cmx180 μm Gemini C18 column for high pH and a 5 μm 4.6x250 mm Gemini C18 column for RP separation to acquire DDA mode data. The DDA data were analyzed, and spectral libraries were generated using MaxQuant’s Andromeda engine (https://www.maxquant.org/). Following corrections to the DIA data, the SWATH-MS target-decoy model was employed to derive quantitative significance. Differential analysis utilizing the MSstats package from the Bioconductor repository (version 4.14.2 https://bioconductor.org/packages/MSstats/) identified proteins with differences >1.2 and p < 0.05 as significant. Functional analyses were then conducted on these proteins.

### 2.8 Enzyme-linked immunosorbent assay

Sixteen pregnant women with hypothyroidism ≤20 weeks’ gestation and 16 normal pregnant women were selected as the validation cohort, and blood samples from this cohort were collected and stored using the same procedure. After thawing the samples at room temperature, selected differentially expressed proteins were verified by sandwich enzyme immunoassay. Human DGKK (DGKK) ELISA kit (Item No. EK10756) produced by Signalway Antibody (USA) was used as the DGKK indicator, and the human S10 calcium-binding protein A8 (S10A8) indicator was verified by the human S100 calcium-binding protein A8 (S100A8) enzyme-linked (22) immunosorbent assay kit (Item No. E-EL-H1289) produced by Wuhan Eliretech Biotechnology Co., Ltd. Plasma was diluted within the linear range of each assay following the manufacturer’s recommendations. Absorbance (OD) was measured at 450 nm using a Biotek EPOCH microplate spectrophotometer. Standard curves were plotted, and protein concentrations were calculated using ELISACALC software.

### 2.9 Statistical analysis

All statistical analyses of the metagenomic and proteomic sequencing data were performed using R (version 3.4.1). The SPSS 26.0 software (IBM Corp. Released 2019.IBM SPSS Statistics for Windows, version 26.0. Armonk, NY: IBM Corp) was used for statistical analysis. Normally distributed measures were described using mean ± standard deviation, t-tests were used for comparisons between groups, medians and quartiles are used to describe non-normally distributed measures, and comparisons between groups were performed using the Wilcoxon rank sum test. Correlation analysis was performed using Spearman’s analysis, and statistical significance was set at P < 0.05.

## 3 Results

### 3.1 Comparison of basic data between the hypothyroid and normal groups

Twenty pregnant women with hypothyroidism and 20 in the normal group during early pregnancy were studied. **Table 1** shows no significant differences in age, BMI, gestational week, GLU, or HGB between groups (p > 0.05).

**Table 1:** Comparison of clinical data between the hypothyroid and normal groups.

	Hypothyroidism group (n=20)	Normal group (n=20)	P-value
age	31.85 ± 4.19	30.90 ± 4.17	0.478
BMI (kg/m^2^)	23.59 ± 3.39	22.41 ± 3.33	0.277
gestation week (week)	15.50 (11.25,16.75)	12.00 (10.25,16.00)	0.429
GLU (g/L)	4.59 ± 0.32	4.67 ± 0.42	0.533
HGB (g/L)	118.30 ± 10.14	117.01 ± 29.10	0.853

p-value less than 0.05 was statistically significant.

### 3.2 Macro gene-based analysis of differential gut microbiota composition and its functional enrichment

**Figure 2:**
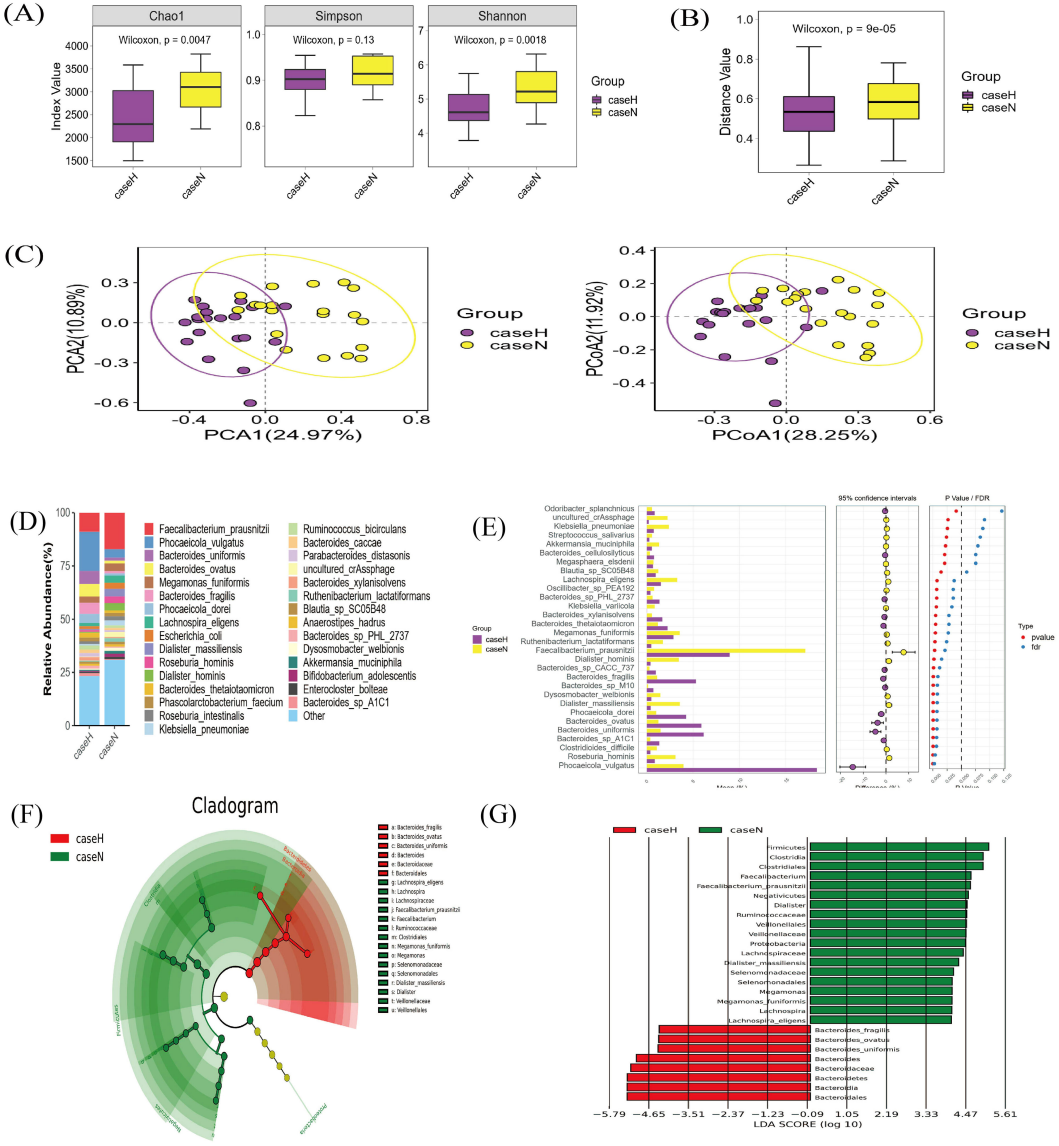
Metagenomic sequencing for species diversity and compositional analysis. **(A)** Alpha diversity analysis: A higher Chao1 index indicates a greater number of species. p < 0.05 indicates statistical significance. **(B)** β-diversity analysis: Based on the β-diversity shown by Bray-Curtis distance analysis; p < 0.05 indicates statistical significance. **(C)** PCA and PCoA plot: PCA1 and PCA2 explained 24.97% and 10.89% of the variance; respectively. 28.25% of the variance, as explained by PCoA1 and 11.92% by PCoA2, respectively. **(D)** Stacked plot of species abundance in the gut microbiota at the species level: Horizontal coordinates represent groupings, and vertical coordinates represent the relative abundance of species. The bar color indicates species classification, and longer lengths indicate higher relative abundances. **(E)** STAMP analysis of the top 30 differentially abundant species between the hypothyroid and normal groups: P-values result from statistical tests and FDR is the false discovery rate, the corrected p-value; points to the left of the dotted line (p < 0.05) denote significant differences. **(F)** LEfSe circular evolutionary branching diagram: This plot identifies the species in both sample groups that best explain the differences between the groups. The inner circle is at a high taxonomic level, and the outer circle is at a low taxonomic level. Each dot represents a specific species classification, with dot size indicating high relative abundance. **(G)** Histogram of LEfSe LDA distribution: This analysis suggests the extent of its influence on intergroup differences. Vertical coordinates represent taxonomic units with differences, and horizontal coordinates represent LDA values. The image shows only classifications with LDA values greater than a set threshold (typically two).

**Figure 3:**
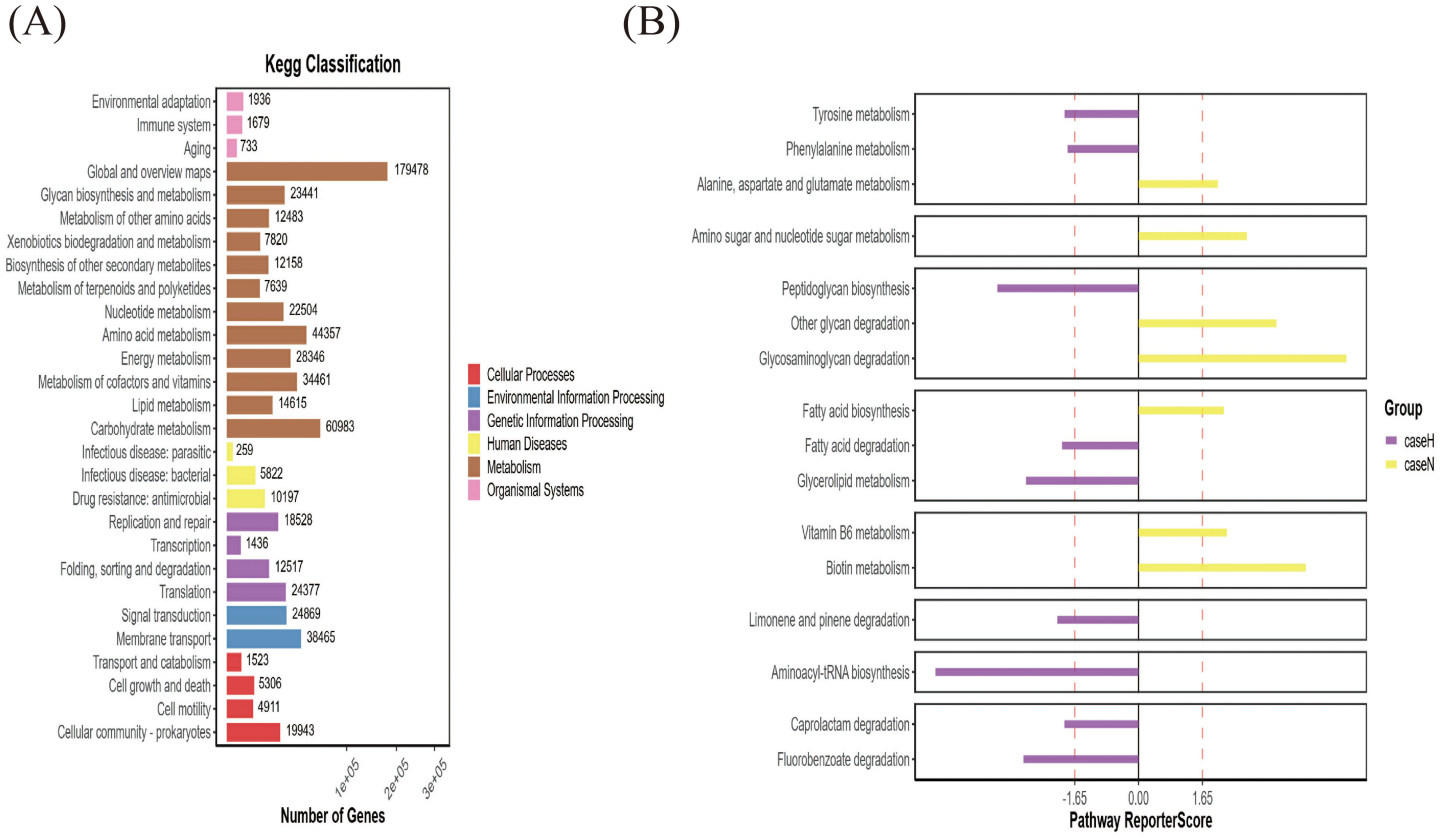
Gene function analysis of gut microbiota. **(A)** Histogram of functional gene statistics. The horizontal axis represents the number of genes, the vertical axis denotes the functional classifications, and the color corresponds to these classifications. The length of the bars reflects the quantity of genes, and a legend on the right side categorizes the primary functional classification for each secondary level. **(B)** Functional KEGG-pathway enrichment map. The horizontal axis displays the Reporter score values, the vertical axis lists the pathways, and the color denotes the subgroup of the enrichment. The diagram only includes functional classifications that exceed a predefined Reporter score threshold.

Analysis of gut microorganism α-diversity showed a significant difference in the chao1 index between the two groups (p < 0.05) (**Figure 2A**). β-diversity, assessed by Bray-Curtis distance, also differed (**Figure 2B**). Principal Component Analysis (PCA) and Principal Co-ordinates Analysis (PCoA) analyses indicated significant differences in species composition (**Figure 2C**), linking dysbiosis in the gut microbiota to the hypothyroidism group. Dominant species in the hypothyroid group included *P. vulgatus* and *B. fragilis* (**Figures 2D, E**). LEfSe analysis identified 27 distinct taxa between groups; *Bacteroidales*, *Bacteroides*, and *B. fragilis* were enriched in the hypothyroid group, while Clostridia prevailed in the normal group. Furthermore, *B. fragilis*, *B. ovatus*, and *B. uniformis* were more abundant in the hypothyroid group, in contrast to *Lachnospira* and *Faecalibacterium prausnitzii*, which were less prevalent (**Figures 2F, G**). KEGG gene pathway analysis revealed active metabolism in carbohydrate, amino acid, glycan biosynthesis, and metabolism (**Figure 3A**). Differential KEGG function analysis annotated genes and their pathways, showing enrichment in peptidoglycan biosynthesis and glycerolipid metabolism in the hypothyroid group (**Figure 3B**).

### 3.3 Serum proteomics sequencing and differential protein function analysis

**Figure 4:**
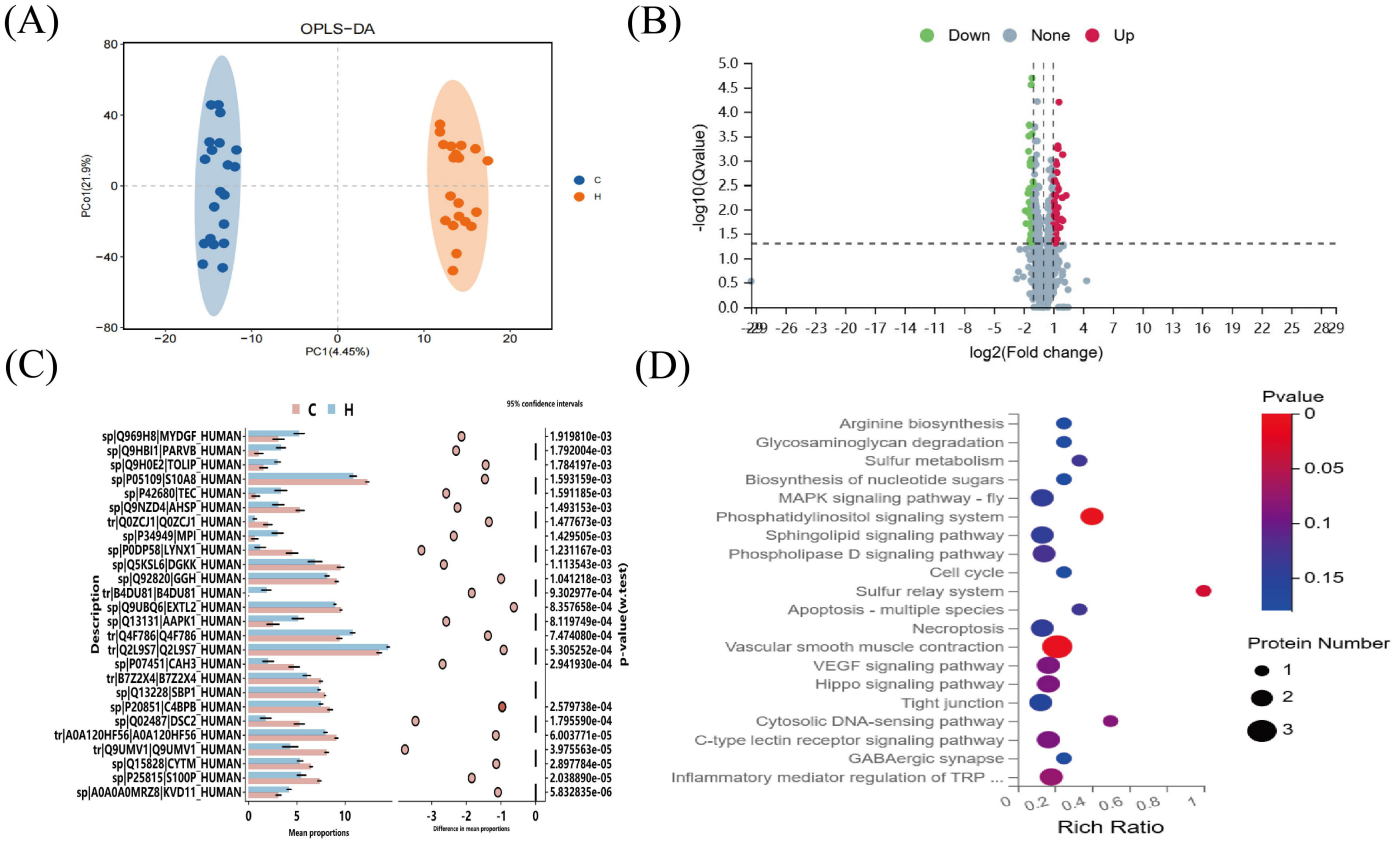
4D-DIA proteomic analysis. **(A)** OPLS-DA Score Chart **(B)** Differential Protein Volcano Plot. The x-axis represents protein differential folds (expressed as log2), while the y-axis shows the corresponding -log10 (Q-value). A Q-value < 0.05 and Fold Change > 1.2 were criteria used to identify significant differential proteins by default. Red points in the graph denote significantly up-regulated proteins, green points indicate significantly down-regulated proteins, and gray points represent proteins with no significant changes. **(C)** SATMP Analysis Plot, displaying the first 26 differentially significant proteins at p < 0.05. **(D)** Differential Protein KEGG-Pathway Enrichment Bubble Plot. The x-axis shows the enrichment ratio, the y-axis labels the KEGG Pathway, and the bubble size reflects the number of proteins annotated to a specific KEGG Pathway. The color gradient indicates the enrichment significance value (p<0.05 denotes statistical significance), with deeper reds signifying smaller significance values.

Orthogonal partial least squares analysis (OPLS-DA) was used to analyze the data from the hypothyroidism and normal groups, demonstrating a statistically significant difference between the two groups (**Figure 4A**). Using 4D-DIA proteomic sequencing, 2196 proteins were screened; 69 proteins were upregulated and 37 downregulated in the hypothyroid group versus the normal group (**Figure 4B**). STAMP analysis indicated significant downregulation of DGKK and S10A8 in the hypothyroid group, both crucial for inflammation and immune response (**Figure 4C**). KEGG pathway analysis revealed significant enrichment in the vascular smooth muscle contraction and phosphatidylinositol signaling pathways (p < 0.05) (**Figure 4D**).

### 3.4 ELISA validation of DGKK and S10A8 expression

**Figure 5:**
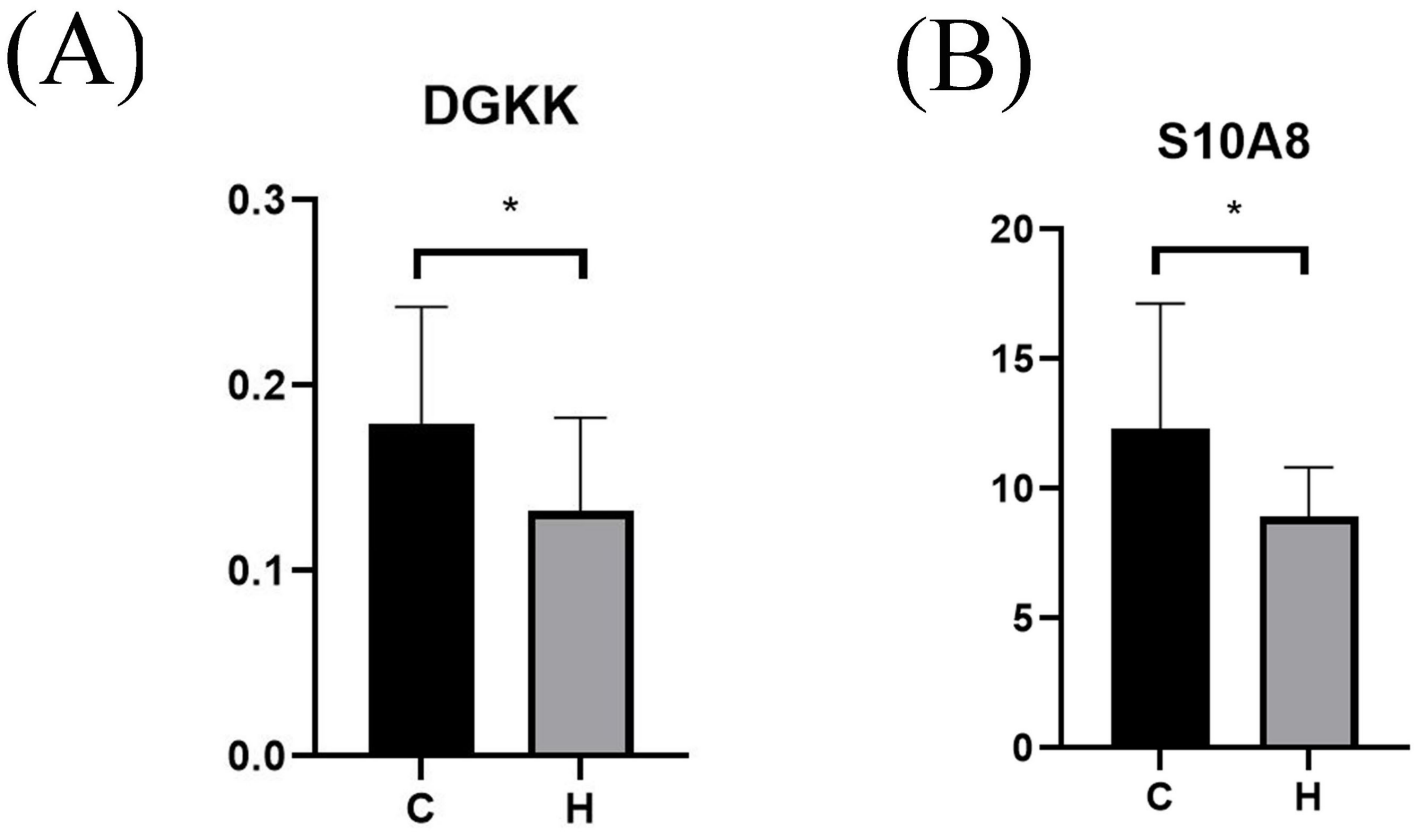
ELISA validation of serum protein level box plots for different populations. Serum levels of DGKK (**Figure 4A**) and S10A8 (**Figure 4B**) in the hypothyroid and normal groups. *p* < 0.05 indicates a statistically significant difference. Group H represents the hypothyroidism group in the first half of pregnancy. Group C represents the normal control group during the same period. * represents P < 0.05.

Serum levels of DGKK and S10A8 in pregnant women with hypothyroidism (Group H) and normal pregnant women (Group C) during pregnancy were determined using ELISA, and as shown in **Figure 5**, serum DGKK and S10A8 were significantly downregulated in the hypothyroidism group compared with the normal group (*p* < 0.05).

### 3.5 Comparison of serologic indices between the hypothyroid and normal groups

**Figure 6:**
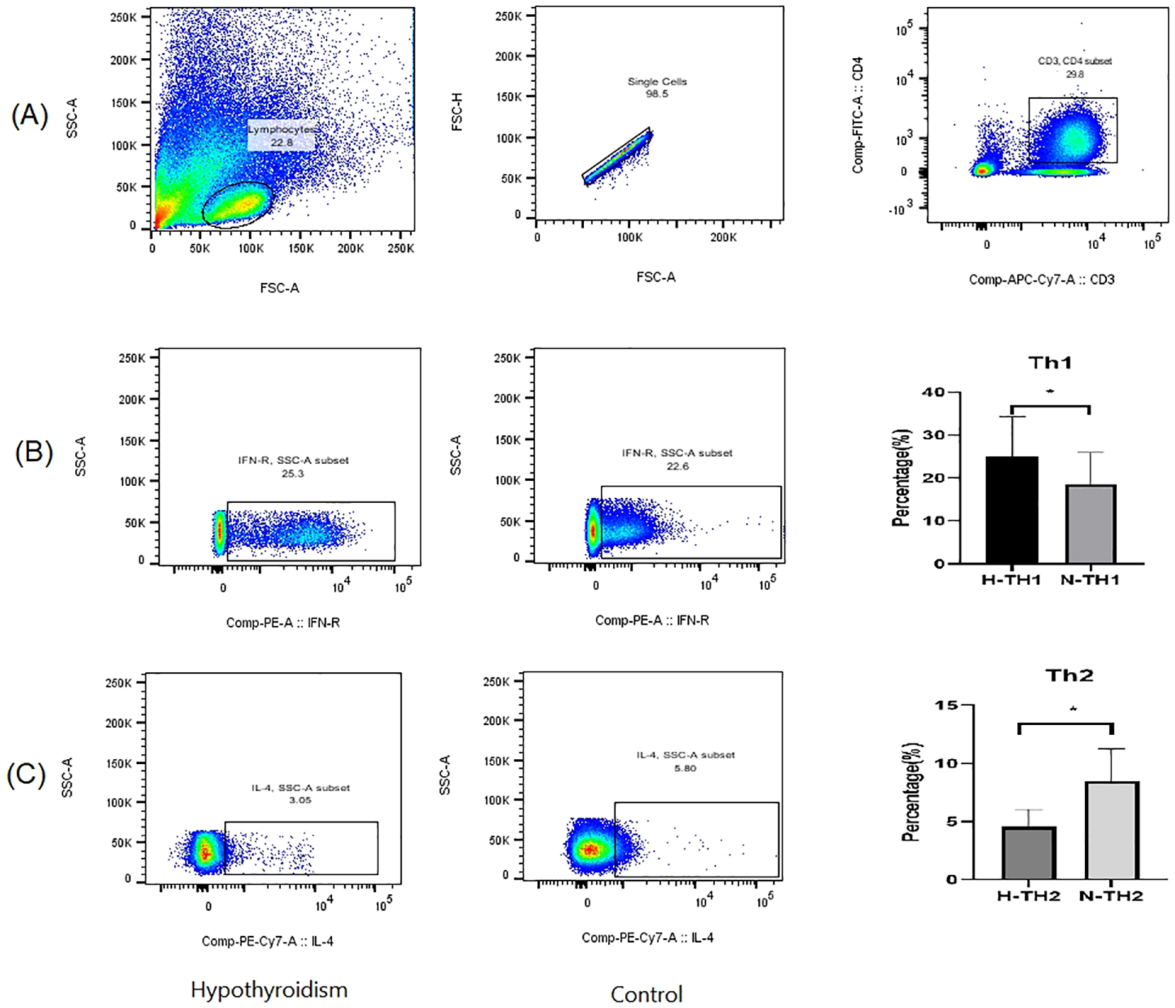
Detection of Th1 and Th2 cells using flow cytometry. **(A)** Flow cytometry gating strategy; **(B)** Percentage of Th1 cells; **(C)** Percentage of Th2 cells. *p* < 0.05 indicates statistical significance. * represents P < 0.05.

Peripheral blood Th1 and Th2 cells were detected in both groups of pregnant women using flow cytometry, and as shown in **Figure 6**, where lymphocytes expressing both CD3 and CD4 were gated as CD4+ T cells. We quantified Th1 and Th2 cells by measuring IFN-γ and IL-4 expression levels. The cytokine levels were determined using the cell microbead array method. As presented in Online Resource 1(**Table 2**), TSH, hs-CRP, IL-2, IL-6, TNF-α, Th1, and Th1/Th2 levels in the hypothyroidism group were significantly higher than those in the normal group, while FT4 and Th2 levels were lower than those in the normal group; the difference between the two groups was statistically significant (*p* < 0.05), while that between the two groups in terms of IL-10 was not.

**Table 2:** Comparison of serologic data between the hypothyroid and normal groups.

	Hypothyroidism group (n=20)	Normal Group (n=20)	P–value
TSH (mIU/L)	5.28 (4.64, 6.66)	1.50 (1.26, 2.41)	<0.026^*^
FT4(mIU/L)	14.1 (11.7, 17.18)	16.45(14.80, 18.95)	0.009^*^
hsCRP	4.01 (3.16, 6.08)	1.52 (0.57, 3.75)	0.001^*^
IL-2	0.63 (0.10, 5.09)	0.06 (0.00, 0.47)	0.007^*^
IL-10	1.06 (0.74, 7.51)	0.98 (0.71, 1.42)	0.355
IL-6	2.64 (1.85, 12.79)	1.44 (1.21, 2.38)	0.038^*^
TNF-α	1.37 (0.39, 10.04)	0.74 (0.05, 1.13)	0.023^*^
Th1	24.92 ± 9.38	18.61 ± 7.34	0.023^*^
TH2	4.55 ± 1.51	8.48 ± 2.77	<0.001^*^
Th1/Th2	6.02 ± 3.12	2.25 ± 0.87	<0.001^*^

p-value less than 0.05 was statistically significant. * represents P < 0.05.

### 3.6 Correlation of dominant strains with screening proteins and serologic indices in the hypothyroid group

**Figure 7:**
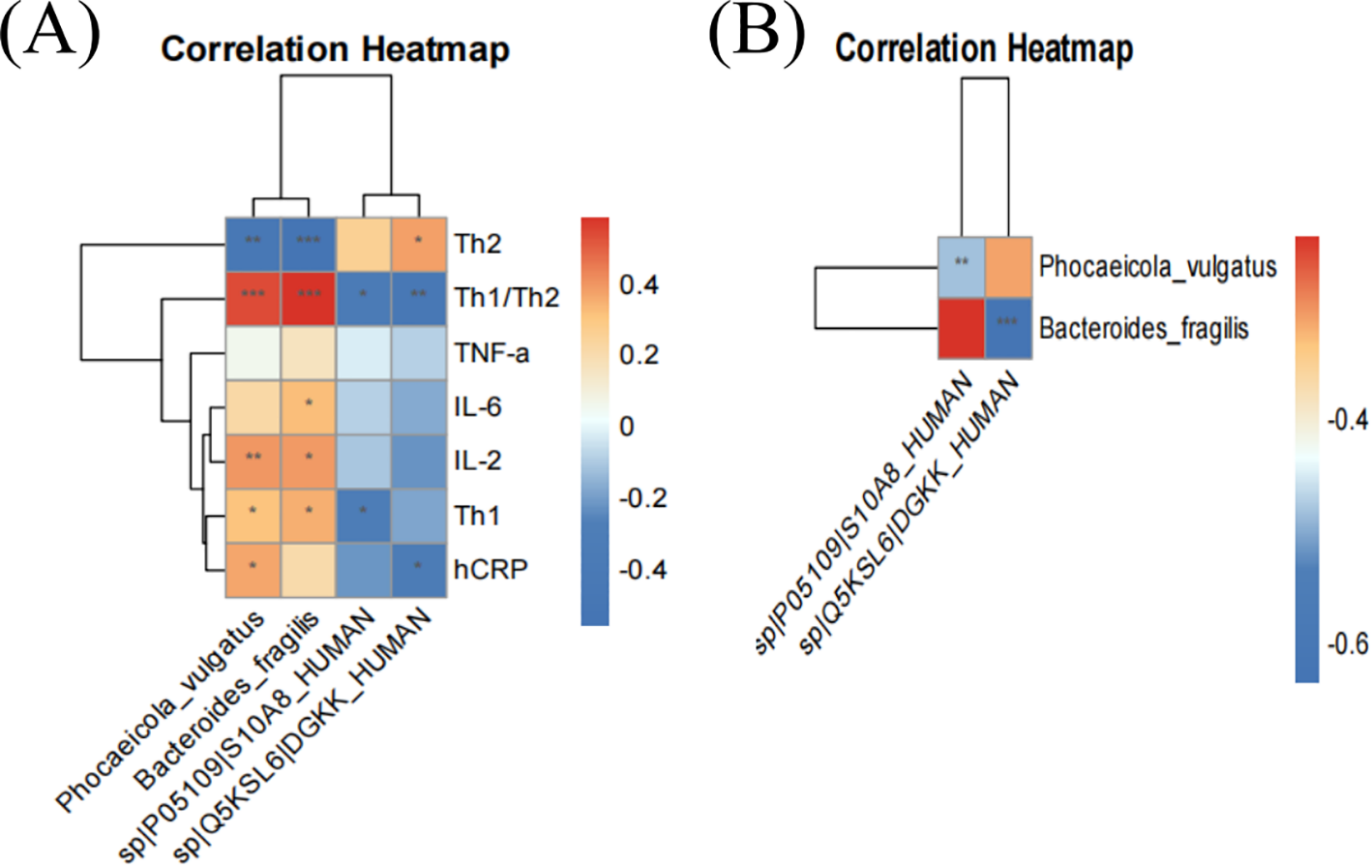
Correlation heatmap of dominant strains with clinical indicators and significantly down-regulated proteins. Red and blue represent positive and negative correlations, respectively. * implies *p* < 0.05, ** implies *p* < 0.01, and *** implies *p* < 0.001.

Spearman’s correlation analysis showed that *P. vulgatus* and *B. fragilis* were significantly positively correlated with Th1/Th2 and positively correlated with IL-2 (**Figure 7A**). DGKK and S10A8 proteins were negatively correlated with Th1/Th2 ratios (**Figure 7A**). *P. vulgatus* was negatively correlated with S10A8, and *B. fragilis* was negatively correlated with DGKK (**Figure 7B**).

## 4 Discussion

Recent years have seen a yearly increase in the incidence of hypothyroidism during pregnancy (1). In the first trimester, the fetus relies primarily on maternal thyroid hormones until the 20th week (2). Notably, the impact of hypothyroidism, including miscarriage, intensifies in the early stages of pregnancy (2). However, the pathogenesis of hypothyroidism and its screening markers in this period remain unclear. Thus, this study employed metagenomic and proteomic approaches to explore their relationship and identify potential hypothyroidism biomarkers in early pregnancy.

This study demonstrated no differences between the hypothyroidism and normal groups in terms of age, BMI, or other general conditions during pregnancy. Metagenomic sequencing results showed that the relative abundances of *P. vulgatus* and *B. fragilis* were higher in the hypothyroidism group, and the outer membrane vesicles of anaplastic bacilli cells were enriched in proteases and peptidoglycans (23, 24). Thus, *P. vulgatus* is potentially pathogenic (25). Moreover, secreted serine and cysteine proteases can induce intestinal inflammation by decreasing trans-epithelial electrical resistance and intestinal mucosal epithelial permeability and allowing innate immune cells such as neutrophils to enter the intestines and destroy the intestinal barrier (26). *B. fragilis* produces enterotoxin and the *B. fragilis* toxin, which activates the IL-17 immune cascade in the intestinal epithelium through nuclear factor kappa-B (NF-κB) and signal transducer and activator of transcription 3 (STAT3) pathways, fostering a pro-inflammatory environment that results in intestinal inflammation (27). Therefore, we hypothesized that both strains contribute to “leaky gut” by exacerbating this inflammation (28), which diminishes the intestinal mucosa’s production and secretion of β-glucuronidase and sulfate lyase, obstructing thyroid hormones’ re-entry into the enterohepatic circulation (29), and prompting an autoimmune response in the thyroid gland (7, 30), leading to hypothyroidism.

In this study, KEGG enrichment analysis revealed significant enrichment of peptidoglycan biosynthesis and glycerol ester metabolism pathways in the hypothyroid group. We hypothesized that *P. vulgatus* and *B. fragilis* contribute to hypothyroidism during early pregnancy due to: (1) an increase in β-N-acetylaminoglucosidase, enhancing peptidoglycan production by catalyzing acetylamino glucose removal from peptidoglycan fragments (31, 32). This stimulates the Nucleotide Binding Oligomerization Domain Containing 1 (NOD1) receptor, triggering the receptor interacting protein kinase 2 (RIP2) kinase and activating the NF-κB pathway (25, 33), which induces an autoimmune response in the thyroid (34); (2) elevated Short-chain fatty acids (SCFA) production resulting from increased mucus-degrading enzymes from *Mycobacterium avium* and intestinal mucus cleavage (35). Furthermore, the AMPK pathway activation increases lysophosphatidic acid production in glycerol ester metabolism, which, via the G Protein-Coupled Receptors (GPCR) pathway, activates the NF-κB pathway (36, 37), escalating pro-inflammatory mediators like TNF-α and IL-6, leading to thyroid autoimmunity and hormonal disruptions.

The proteomic results of this study showed that DGKK and S10A8 were significantly down-regulated in patients with hypothyroidism in the first half of pregnancy. ELISA validation showed that DGKK and S10A8 were significantly down-regulated in patients with hypothyroidism in the first half of pregnancy. The down-regulation of DGKK affects the protein kinase C pathway and reduces the H2O2 production (38). H2O2 is synthesized by dual oxidase (DUOX) and participates in the catalysis of iodide by thyroid peroxidase (TPO) (39, 40). As an essential molecule for iodine binding and thyroid hormone synthesis, a decrease in H2O2 levels affects iodine binding in thyroid cells and the synthesis and secretion of thyroid hormones (41). Downregulation of S10A8 inhibits NADPH oxidase and reduces the production of H2O2 (42–44). S10A8 also affects Adenosine triphosphate (ATP) production, regulates Ca2+ reduction, influences phosphatidylinositol (PI) and H2O2 production (45), and is involved in the development of hypothyroidism during the first half of pregnancy.

Enrichment analysis of differential protein KEGG pathways in this study showed that phosphatidylinositol signaling and smooth vascular muscle contraction pathways were significantly enriched. Therefore, we hypothesized that the downregulation of DGKK and S10A8 is involved in the development of hypothyroidism in the first half of pregnancy, which may (1) reduce Diacylglycerol (DAG) phosphorylation and inhibit phosphatidic acid production by the protein kinase C pathway by downregulation of DGKK (46), which reduces PI in the phosphatidylinositol signaling pathway (45) and in turn affects inositol-dependent thyroid stimulating hormone (TSH) signaling (47), resulting in altered TSH synthesis and secretion and leading to hypothyroidism (48) (2). S10A8 downregulation affects ATP and Ca2+, not only vascular smooth muscle contraction (49, 50), but also regulates thyroglobulin (thyroid hormone precursor) synthesis and secretion in thyroid cells (51), promoting the onset of hypothyroidism.

Flow cytometry results indicated an increase in Th1 cells and a decrease in Th2 cells in pregnant women with hypothyroidism compared to the normal group, with a Th1/Th2 balance shift toward Th1. Additionally, hs-CRP, IL-2, IL-6 and TNF-α levels were elevated in the hypothyroidism group, suggesting an inflammatory response in these patients during the first half of pregnancy, aligning with previous studies (12). When the Th1/Th2 ratio is elevated, Th1 dominates the immune response, which not only promotes the increased release of IL-2, IL-6, TNF-α (52, 53), but also affects autoimmunity and stimulates the synthesis and release of chemotactic immunity factors from thyroid cells, thereby expanding autoimmune feedback (54–56) and accelerating the Fas-mediated apoptosis of thyroid cells (54). This promotes thyroid damage and induces hypothyroidism (52).

The correlation analysis indicated that *P. vulgatus* and *B. fragilis* significantly correlate with Th1/Th2 ratios, showing positive associations with IL-2, DGKK, and S10A8, yet negative associations with Th1/Th2. *P. vulgatus* negatively correlates with S10A8, while *B. fragilis* negatively correlates with IL-2 and DGKK, suggesting distinct bacterial-protein interactions. These interactions are believed to contribute to hypothyroidism by altering Th1/Th2 balance and cytokine profiles through several mechanisms: (1) increased *B. fragilis* may enhance protease release, acting as virulence factors to disrupt the intestinal barrier and degrade extracellular DGKK (46) and S10A8; (2) upregulation of *Bacteroides* may modulate transcription factors, increasing Th1 and decreasing Th2 cells, thereby shifting the Th1/Th2 balance and impacting pro-inflammatory cytokine release (57); (3) downregulation of S10A8 impairs myeloid-derived suppressor cells (MDSC) via the Toll-like receptor 4 (TLR4) pathway (58, 59), thus hindering T cell-mediated immunity and disrupting Th1/Th2 balance (60, 61). These pathways potentially elevate Th1/Th2 ratios, exacerbate thyroid damage, and induce hypothyroidism.

Our study has limitations. The small sample size, influenced by regional dietary and lifestyle habits may have impacted our results. Secondly, due to limitations in sample size, our power analysis reveals that the current study achieves approximately 70% statistical power for detecting large effect sizes. Thus, further research with a larger sample and diverse histological approaches is necessary to investigate hypothyroidism’s onset during early pregnancy.

In summary, we examined the altered gut microbiota and proteomic characteristics in patients with early pregnancy hypothyroidism and investigated their association with Th1/Th2 cells using macro proteomics. This was an exploratory study. Our findings suggest that increased *P. vulgatus* and *B. fragilis*, decreased DGKK and S10A8 proteins, and a left shift in the Th1/Th2 balance in patients with hypothyroidism in the first half of pregnancy may be associated with the development of the disease. This study offers novel insights into early pregnancy hypothyroidism pathogenesis and potential biomarker identification.

## Data Availability

Metagenomics and mass spectrometry proteomics data have been deposited in the ProteomeXchange Consortium (https://proteomecentral.proteomexchange.org) with dataset identifier PXD055369. Other data were deposited in Figshare database ((https://figshare.com), dataset DOI is 10.6084/m9.figshare.26879455.
