# Supplementary material for: An exploratory study on the metagenomic and proteomic characterization of hypothyroidism in the first half of pregnancy and correlation with Th1/Th2 balance
Source: Front Immunol. 2025 May 15;16:1500866. doi: 10.3389/fimmu.2025.1500866 (PMC12120637; doi:10.3389/fimmu.2025.1500866)
Supplement: Supplementary file 1 [file DataSheet1.docx]

Supplementary Material

**Supplementary Figures**


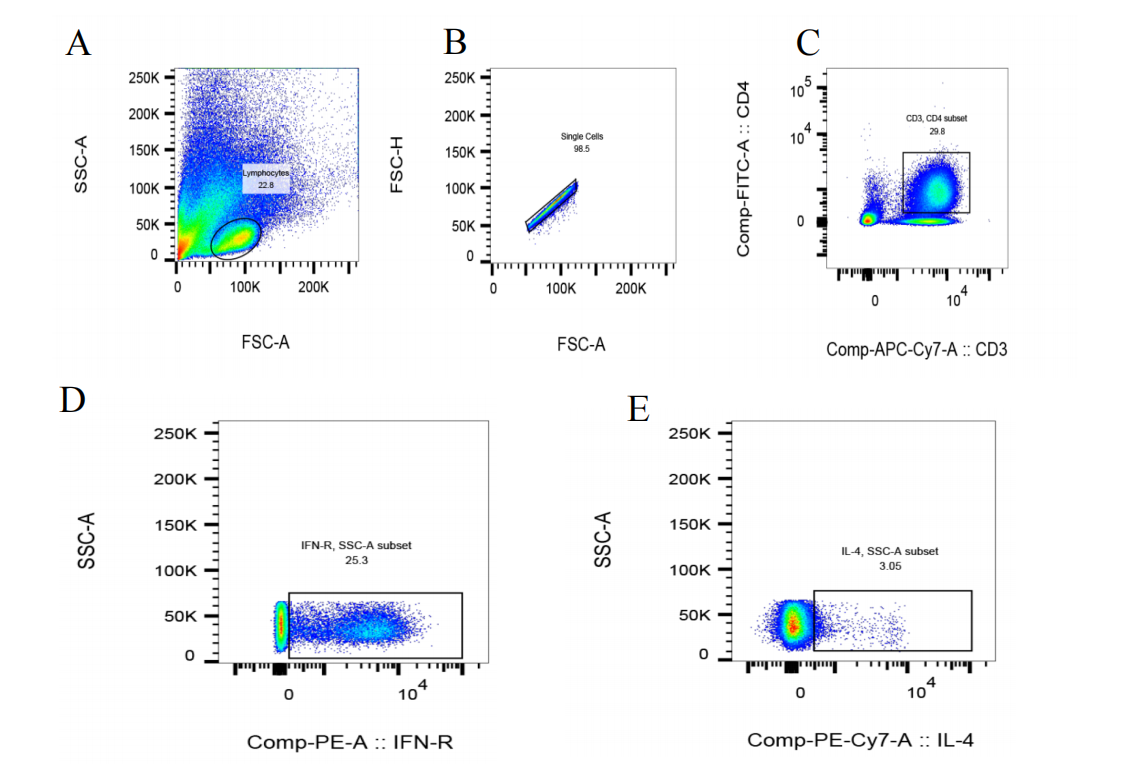


**Supplementary Fig.1** Th1 and Th2 Cell Gating Strategy

The gating strategy was implemented following compensation controls. Initial lymphocyte population identification was performed based on cellular characteristics, utilizing forward scatter (FSC) and side scatter (SSC) parameters plotted on the x-axis and y-axis, respectively (Figure 1A). Subsequent to this primary gating, single-cell populations were isolated through the exclusion of doublets, achieved by plotting forward scatter height (FSC-H) against forward scatter area (FSC-A) on the y-axis and x-axis, respectively (Figure 1B). CD4+ T cell populations were then specifically gated using APC-CY7 and FITC fluorescence parameters on the x-axis and y-axis (Figure 1C). For quantitative analysis of T helper cell subsets, Th1 and Th2 cell populations were identified through the detection of intracellular cytokines, with IFN-γ and IL-4 expression plotted against SSC on the y-axis and x-axis, respectively, representing the proportions of CD3+CD4+IFN-γ+ Th1 cells and CD3+CD4+IL-4+ Th2 cells (Figure 1D, E). All cellular proportions and corresponding graphical representations were systematically recorded, with data analysis performed using FlowJo software (Tree Star, Ashland, OR, USA，version10.8.1).

**
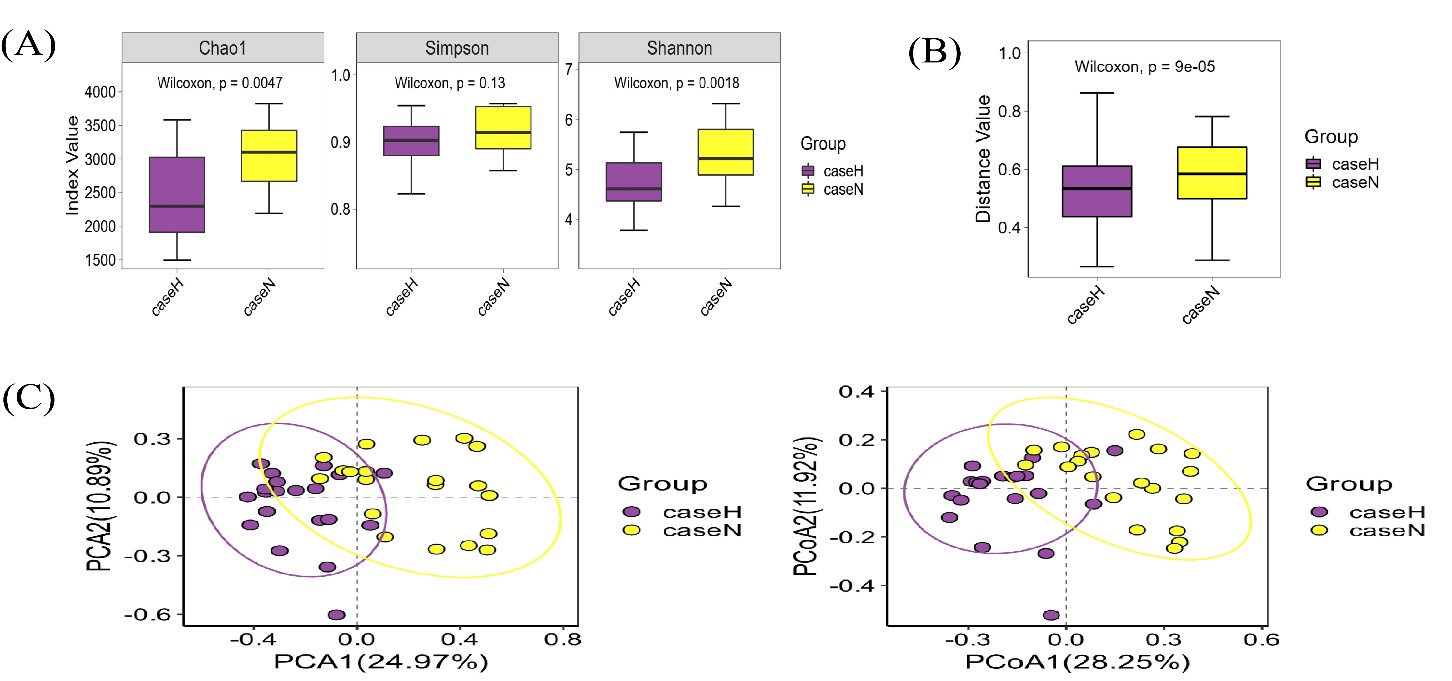

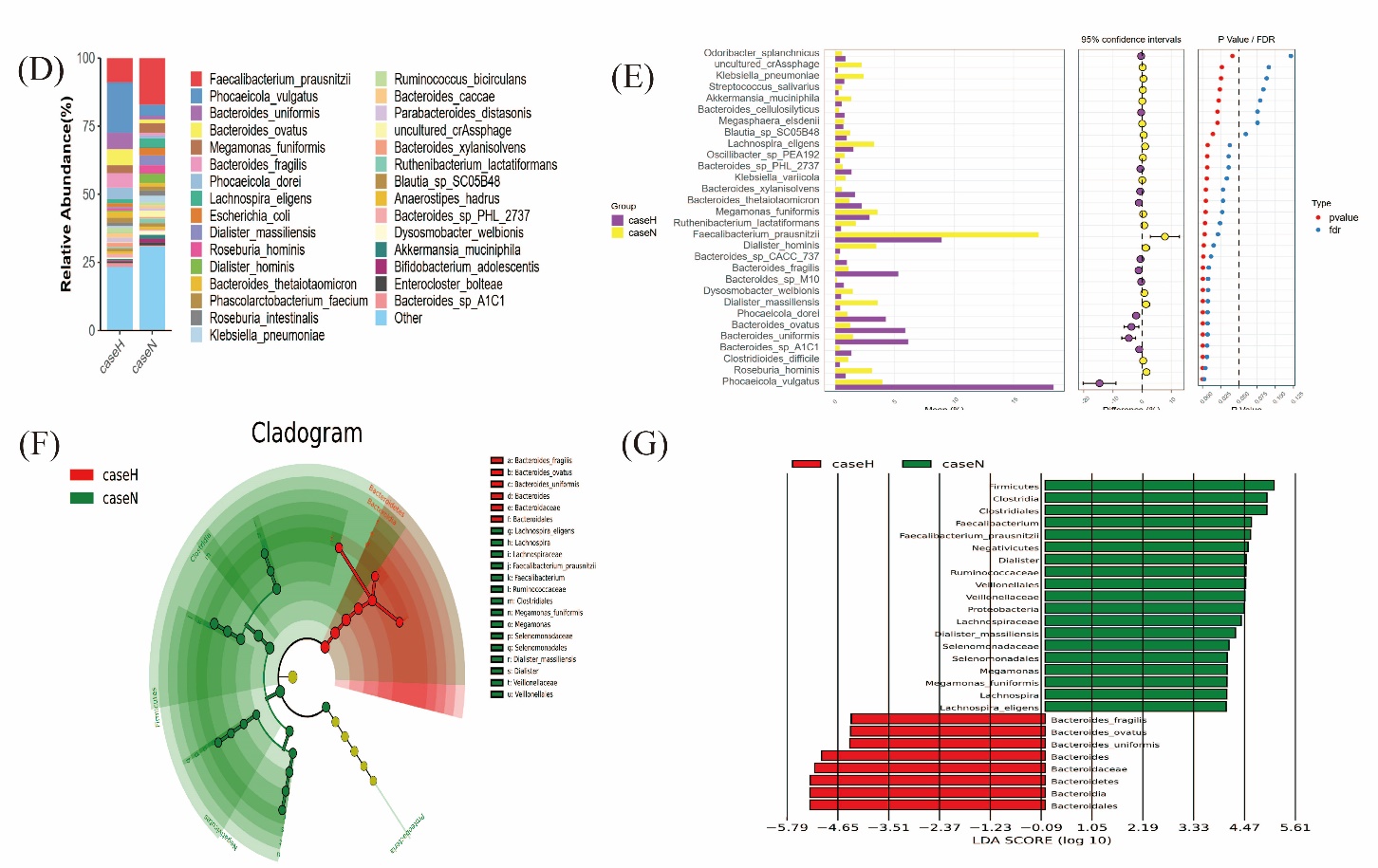
**

**Supplementary Fig.2** Metagenomic sequencing for species diversity and compositional analysis.

(A) Alpha diversity analysis: A higher Chao1 index indicates a greater number of species.p < 0.05 indicates statistical significance. (B) β-diversity analysis: Based on the β-diversity shown by Bray-Curtis distance analysis; p < 0.05 indicates statistical significance. (C) PCA and PCoA plot: PCA1 and PCA2 explained 24.97% and 10.89% of the variance; respectively. 28.25% of the variance, as explained by PCoA1 and 11.92% by PCoA2, respectively. (D) Stacked plot of species abundance in the gut microbiota at the species level: Horizontal coordinates represent groupings, and vertical coordinates represent the relative abundance of species. The bar color indicates species classification, and longer lengths indicate higher relative abundances. (E) STAMP analysis of the top 30 differentially abundant species between the hypothyroid and normal groups: P-values result from statistical tests and FDR is the false discovery rate, the corrected p-value; points to the left of the dotted line (p < 0.05) denote significant differences. (F) LEfSe circular evolutionary branching diagram: This plot identifies the species in both sample groups that best explain the differences between the groups. The inner circle is at a high taxonomic level, and the outer circle is at a low taxonomic level. Each dot represents a specific species classification, with dot size indicating high relative abundance. (G) Histogram of LEfSe LDA distribution: This analysis suggests the extent of its influence on intergroup differences. Vertical coordinates represent taxonomic units with differences, and horizontal coordinates represent LDA values. The image shows only classifications with LDA values greater than a set threshold (typically two).

**
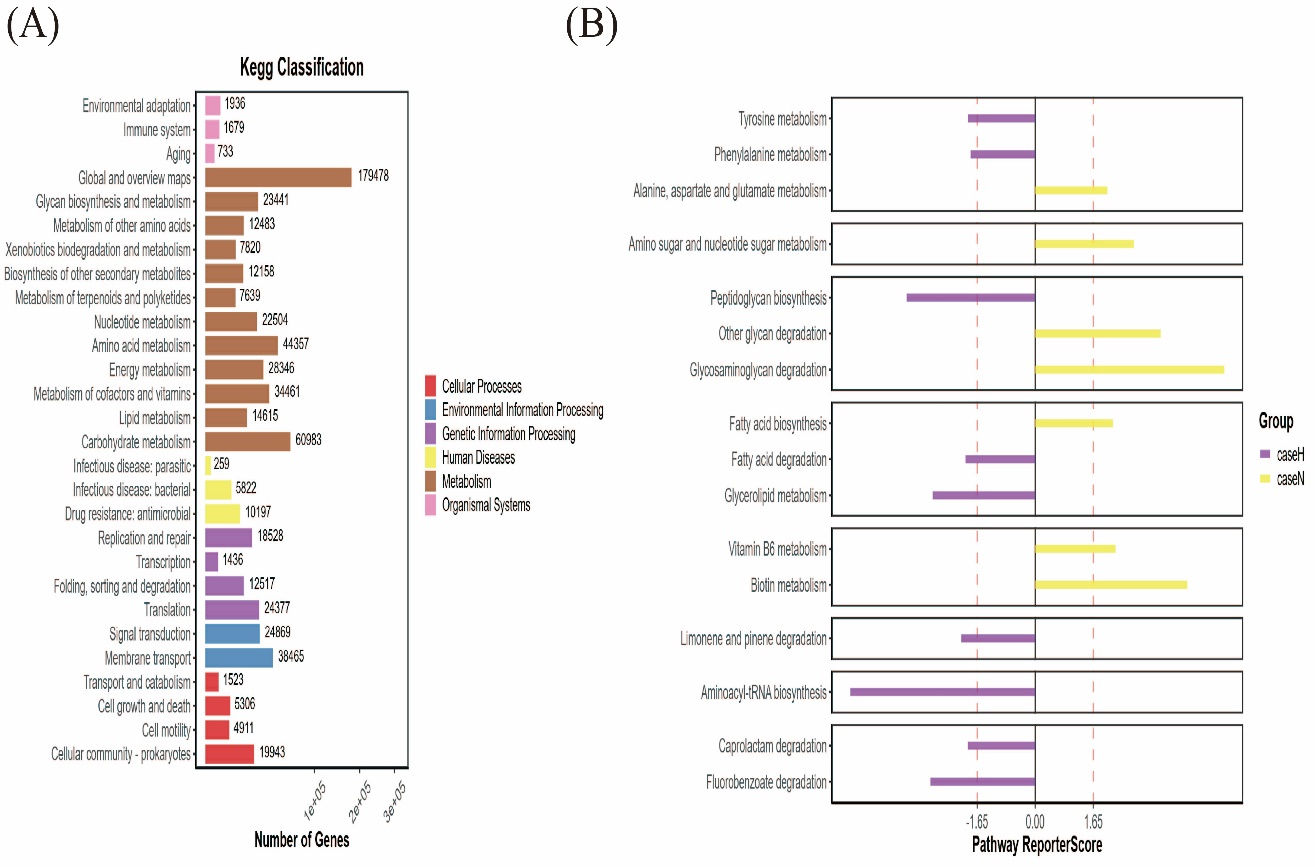
Supplementary Fig. 3** Gene function analysis of intestinal flora

(A) Histogram of functional gene statistics. The horizontal axis represents the number of genes, the vertical axis denotes the functional classifications, and the color corresponds to these classifications. The length of the bars reflects the quantity of genes, and a legend on the right side categorizes the primary functional classification for each secondary level. (B) Functional KEGG-pathway enrichment map. The horizontal axis displays the Reporter score values, the vertical axis lists the pathways, and the color denotes the subgroup of the enrichment. The diagram only includes functional classifications that exceed a predefined Reporter score threshold.

**
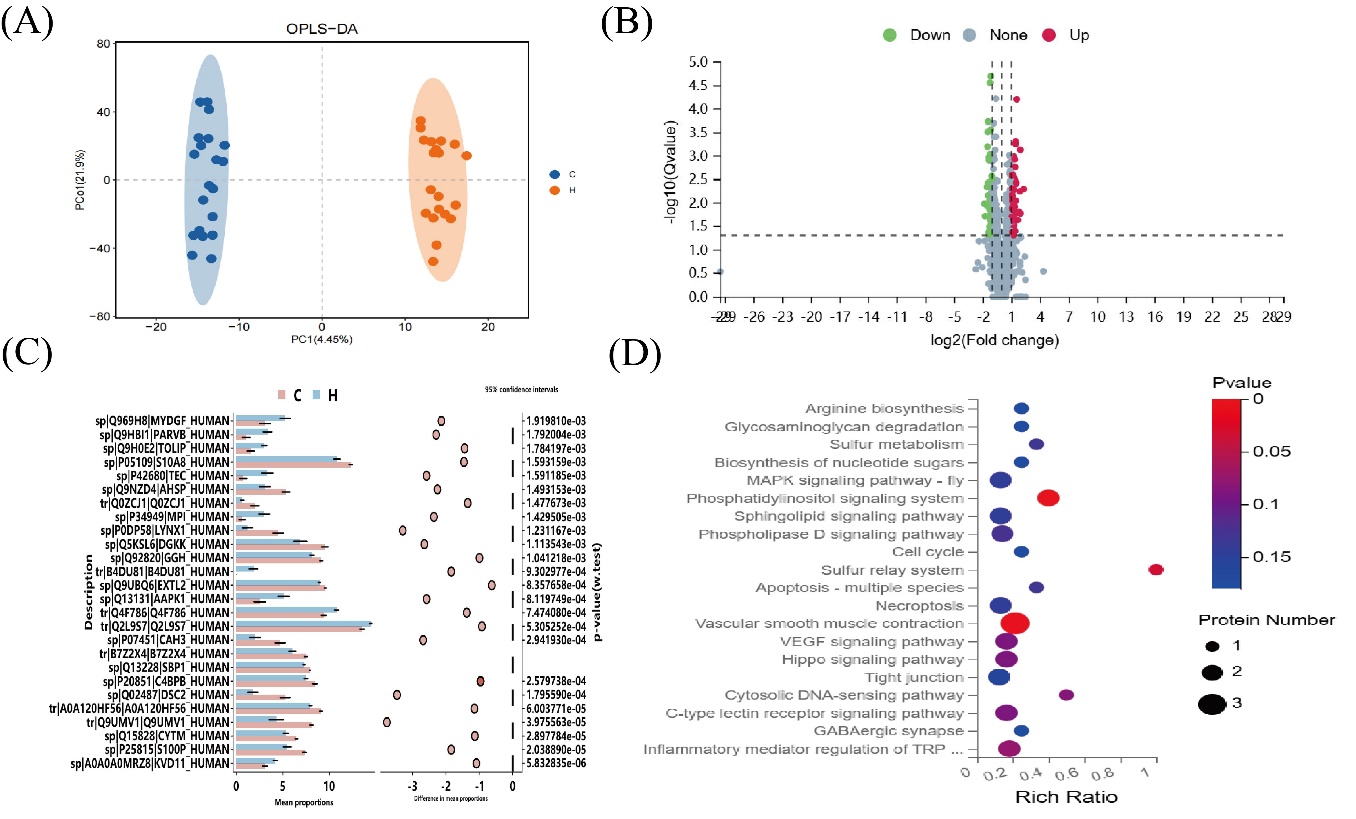
Supplementary Fig. 4** 4D-DIA Proteomic Analysis

(A) OPLS-DA Score Chart (B) Differential Protein Volcano Plot. The x-axis represents protein differential folds (expressed as log2), while the y-axis shows the corresponding -log10 (Q-value). A Q-value < 0.05 and Fold Change > 1.2 were criteria used to identify significant differential proteins by default. Red points in the graph denote significantly up-regulated proteins, green points indicate significantly down-regulated proteins, and gray points represent proteins with no significant changes. (C) SATMP Analysis Plot, displaying the first 26 differentially significant proteins at p < 0.05. (D) Differential Protein KEGG-Pathway Enrichment Bubble Plot. The x-axis shows the enrichment ratio, the y-axis labels the KEGG Pathway, and the bubble size reflects the number of proteins annotated to a specific KEGG Pathway. The color gradient indicates the enrichment significance value (p<0.05 denotes statistical significance), with deeper reds signifying smaller significance values.

**
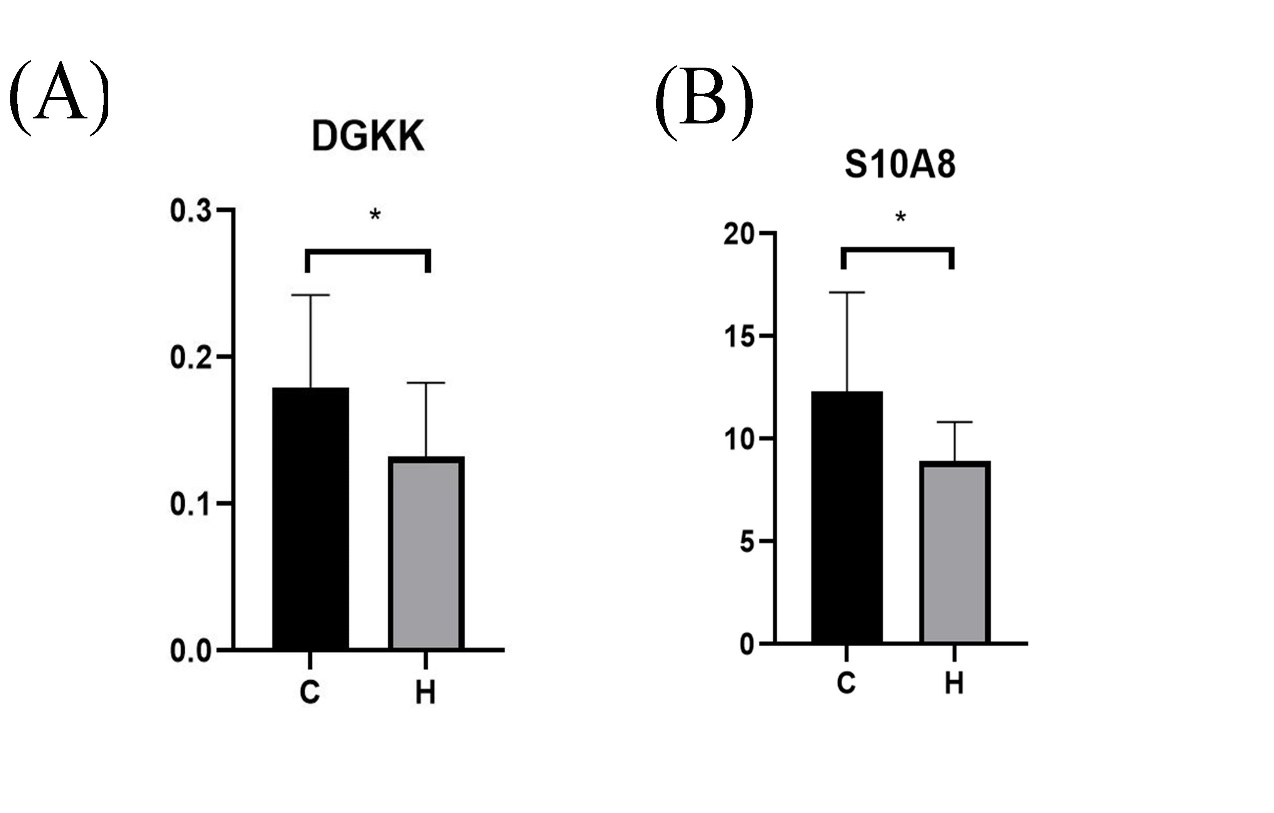
**

**Supplementary Fig. 5** ELISA validation of serum protein level box plots for different populations

Serum levels of DGKK (Fig 4A) and S10A8 (Fig 4B) in the hypothyroid and normal groups. *p* < 0.05 indicates a statistically significant difference.

Group H represents the hypothyroidism group in the first half of pregnancy.

Group C represents the normal control group during the same period.


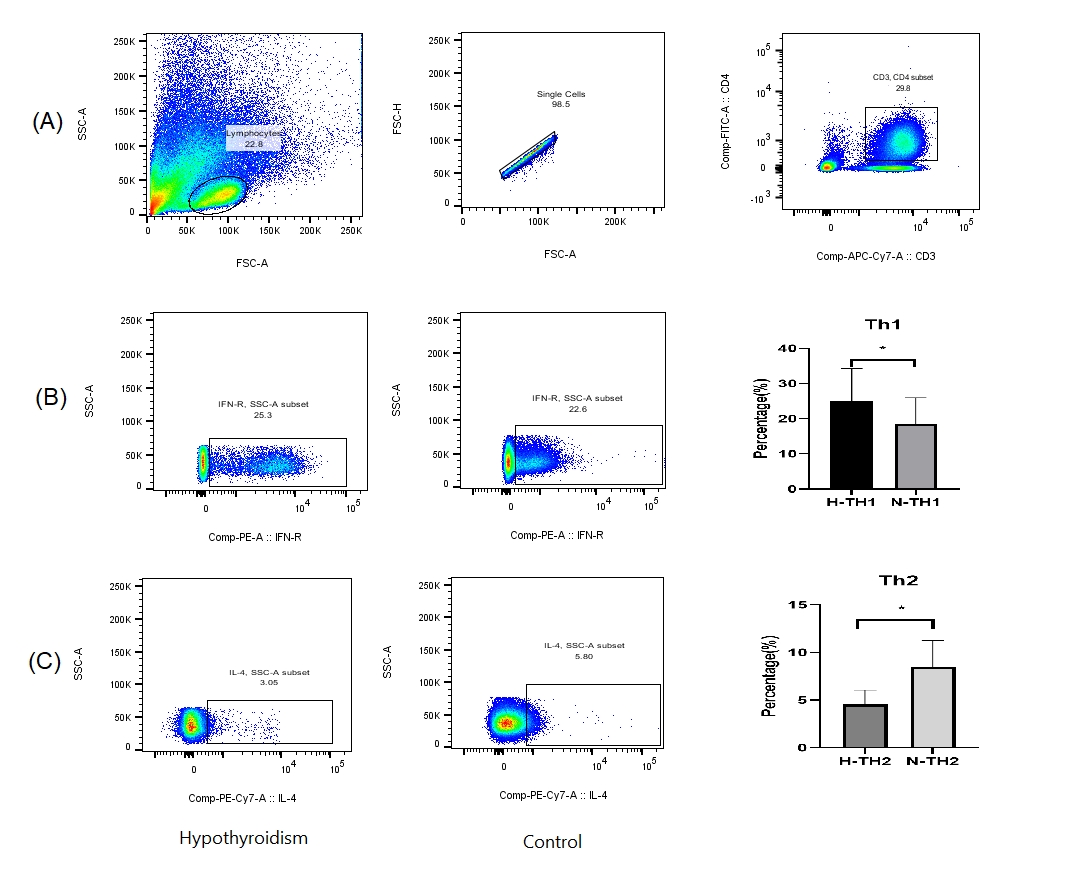


**Supplementary Fig. 6** Detection of Th1 and Th2 cells using flow cytometry. (A) Flow cytometry gating strategy; (B) Percentage of Th1 cells; (C) Percentage of Th2 cells. *p* < 0.05 indicates statistical significance.


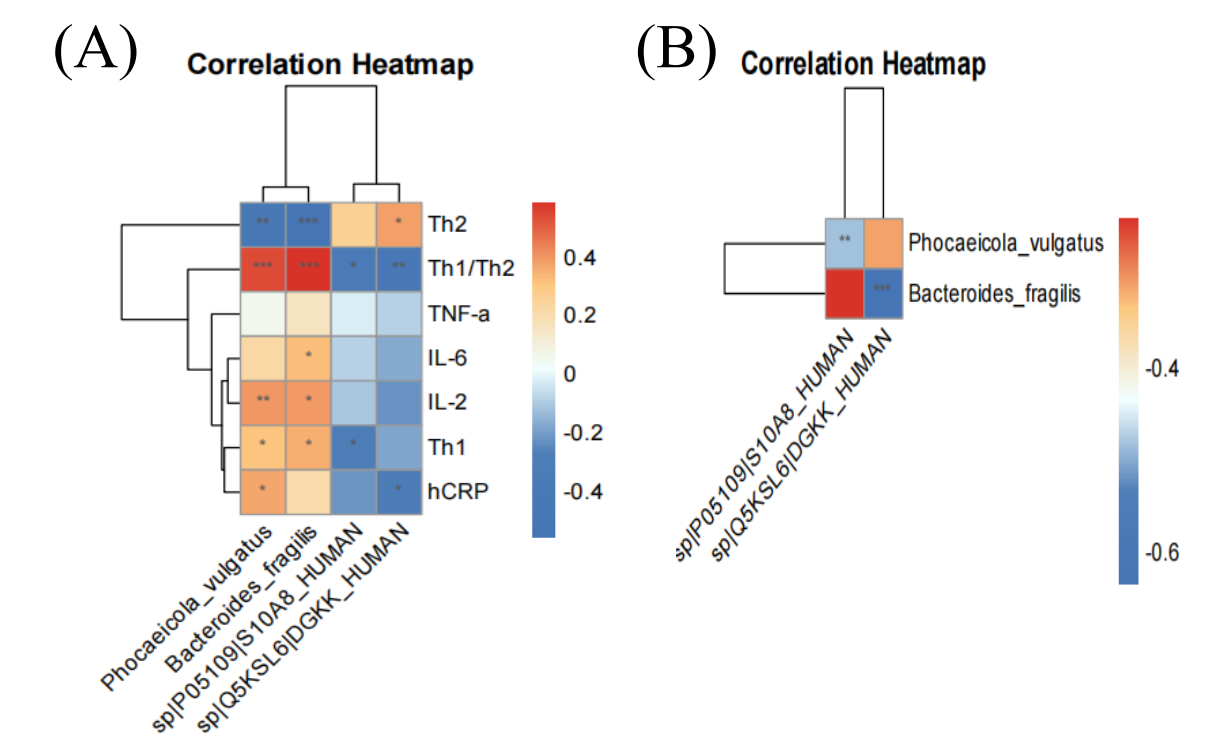


**Supplementary Fig. 7** Correlation heatmap of dominant strains with clinical indicators and significantly down-regulated proteins

Red and blue represent positive and negative correlations, respectively. * implies *p* < 0.05, ** implies *p* < 0.01, and *** implies *p* < 0.001.
